# The Role of *Fusobacterium* in Periodontal Disease and Its Implications for Cardiovascular Health

**DOI:** 10.3390/biomedicines14030697

**Published:** 2026-03-17

**Authors:** Yvonne Prince, Glenda Mary Davison, Tandi Matsha, Shanel Raghubeer

**Affiliations:** 1 SAMRC/CPUT Cardiometabolic Health Research Unit, Department of Biomedical Sciences, Faculty of Health and Wellness Science, Cape Peninsula University of Technology, Cape Town 7530, Western Cape, South Africa; princey@cput.ac.za (Y.P.); davisong@cput.ac.za (G.M.D.); tandi.matsha-erasmus@smu.ac.za (T.M.); 2 Sefako Makgatho Health Science University, GaRankuwa, Pretoria 0208, Gauteng, South Africa

**Keywords:** *Fusobacterium nucleatum*, periodontal disease, oral microbiome, cardiovascular disease, systemic inflammation, microbial imbalances

## Abstract

*Fusobacterium* species, particularly *Fusobacterium nucleatum*, is known as a key adhesive bridging microorganism in the development of periodontal disease, inducing microbial imbalances and chronic inflammation within the oral cavity. Their role is to provide a bridge between both early colonisers (such as Streptococcus and Actinomyces) and late colonisers (such as *Porphyromonas gingivalis* and *Treponema denticola*), which results in multispecies biofilm formation. This triggers an immune reaction which may provide both a protective and destructive effect on the periodontal ligament and alveolar bone. Recent studies have discovered their significance beyond oral pathology. Therefore, *Fusobacterium* have been implicated in several systemic diseases, including cardiovascular disease (CVD). Virulent mechanisms, such as adhesion, invasion, modulation of host immunity, and pro-inflammatory signalling, contributes to periodontal tissue bone loss and entry into the circulation. Circulating bacteria interact with vascular endothelium and promote atherosclerotic plaque formation. The role of *Fusobacterium nucleatum* as a microbial link between periodontal disease and cardiovascular disease is highlighted and discussed. Overall, current evidence is mostly observational and preclinical, supporting an associative link between *F. nucleatum*-mediated periodontal disease and CVD. The literature highlights key mechanistic pathways while underscoring the important need for longitudinal studies to clarify causality and identify target therapeutic interventions.

## 1. Introduction

The oral microbiome plays an important supporting role in maintaining homeostasis, impacting not only oral health, but also contributing to the development of systemic comorbidities [1,2]. It is highly diverse, comprising more than 700 bacterial species in multiple niches, such as tongue, cheeks, teeth, gums, and saliva. Although each niche supports different oral microbial communities, core species are shared across the habitats [1]. Key bacterial phyla include Firmicutes, Bacteroidetes, Proteobacteria, Actinobacteria, Fusobacteria, and Spirochaetes, which serve as a protective mechanism to maintain oral health and prevent systemic comorbidities [3]. These microbes survive through biofilm formation, which serve an ecological function that can be both beneficial and potentially harmful [1]. Microbes develop adaptive mechanisms that allow them to survive, colonise, and maintain their function [4]. If the oral microbiome balance is disturbed, harmful oral pathogens may become dominant and cause disease, such as dental caries and periodontal disease, which may result in systemic comorbidities if left untreated [2].

Oral disease, such as dental caries, gingivitis, and periodontitis develop through a complex interaction between oral bacteria in the mouth, the host immune system, and lifestyle factors [2]. Oral bacteria, such as *Streptococcus mutans*, in the form of biofilms (dental plaque) [5] adhere to areas (such as teeth) when excessive amounts of sugar are consumed by the host [6]. These oral bacteria produce acids that lower the pH in the mouth, leading to the destruction of teeth enamel and the development of cavities (dental caries) [6]. If there is an overgrowth of these bacteria along the gumline, it results in an inflammatory response, resulting in gingivitis. Gingivitis is known to cause gum bleeding, and, if untreated, the plaque may harden into tartar, allowing pathogenic bacteria to invade deeper into the gums destroying tissue connected to bones, thereby progressing to periodontitis [7]. Risk factors, such as poor oral hygiene and diet (sugars), smoking, and unfamiliar systemic comorbidities, may further increase the risk of disease progression [2].

Periodontitis is a periodontal chronic oral disease, recognised as an inflammatory condition, and is cited as the 6th most common non-communicable infection that occurs due to an imbalance between the host commensal oral microbiome and the immune response, resulting in oral tissue destruction and overgrowth of pathogenic bacteria [8]. Recent studies have indicated that periodontitis is not only caused by one type of bacteria, but by various bacteria working in synergy [9]. While research has traditionally focused on the harmful “red complex” bacteria, the recent model of polymicrobial synergy and dysbiosis has provided a better understanding of the condition. For instance, red complex bacteria, such as *Porphyromonas gingivalis*, disrupt the immune system and cause inflammation [10]. Inflammation stimulates overgrowth of other harmful bacteria, causing damage to the gum and supporting tissues. Thus, treating and preventing oral and systemic comorbidities requires a dual focus on oral bacteria and the overall host response [11].

Reports have shown that oral disease affects low-, middle-, and high-income countries, resulting in lost productivity and compromised quality of life [12]. In 2021, the 74th World Health Assembly of the World Health Organization (WHO) recognised and adopted the resolution on oral health highlighting the critical role of oral well-being in overall health. The WHO have therefore planned prevention and control methodologies to assist in the resolution of the condition [12,13]. Both periodontitis and CVDs have been identified as major health burdens [14]. CVDs are the leading cause of death globally and have been associated with 17.9 million annual deaths [15]. Chronic inflammation is an underlying complication in both periodontitis and atherosclerosis; thus, it is hypothesised that periodontal inflammation may contribute to endothelial dysfunction and the promotion of atherosclerosis. Current systematic reviews and meta-analyses report that periodontitis is associated with an 84% increased risk of acute myocardial infarction (AMI), independent of traditional cardiovascular risk factors, with greater periodontal disease severity correlating with higher AMI risk [16].

In support of this, both *Fusobacterium nucleatum* and *Porphyromonas gingivalis* have been detected in these diseases [17]. Furthermore, research has shown that treatment of periodontitis reduces systemic inflammation, improves endothelial function, and reduces the risk of atherosclerosis [18].

In individuals with a healthy oral microbiome, Gram-positive cocci and bacilli dominate as early colonisers, while Gram-negative anaerobic rods, such as *F. nucleatum*, bridge bacterial coaggregation [19]. *Fusobacterium nucleatum* plays a key role in driving the pathogenic shift that contributes to periodontal disease and inflammation, acting as a bridge between early colonisers, such as *Streptococcus* species, and late colonisers, such as *Porphyromonas gingivalis* and *Aggregatibacter actinomycetemcomitans* [20]. Researchers have observed that *F. nucleatum* produces a corn cob-like structure when co-cultured with *S. sanguinis*, while multiple *S. sanguinis* cells attach to a single *F. nucleatum* cell [21]. This is due to the rod shape of *F. nucleatum*, which allows the structural relationship necessary for polymicrobial biofilm formation and interaction between early and late colonisers [22].

Although the link between periodontitis and cardiovascular diseases is well established, most research has focused on “red complex” bacteria, such as *Bacterioides*; however, less is known about the role of *Fusobacterium* [14]. A literature search was performed using PubMed, Google scholar, and Science Direct databases up to the end of 2025. Articles relevant to the subject were analysed to collect the most recent evidence, identify connections, and emphasise areas for future investigation. This review aims to critically evaluate the current literature on the role of the *Fusobacterium* species in systemic inflammation and the development of CVD. The focus of this review will be on the microbiology of *Fusobacterium*, the mechanisms by which *Fusobacterium* contributes to endothelial dysfunction, and atherogenesis, as well as highlighting current diagnostic and therapeutic strategies.

## 2. Microbiology of *Fusobacterium*

With the growing use of omics technologies, it has become evident that our understanding of the microbial domain is limited, given the diversity of unexamined oral bacteria and their potential roles in human disease and the environment [23,24]. *Fusobacteria* grow anaerobically and belong to the phylum Fusobacteriota (previously known as Firmicutes). These genera are found in the oral cavity of the mouth and other mucosal sites [22]. Several species of Fusobacteria have been identified, of which *F. nucleatum* and *F. periodonticum* have been identified in the oral cavity and subgingival plaque of humans and have been associated with CVDs and periodontitis [16,25,26]. Their presence in normal anatomical sites suggest that they are part of the resident commensal microbiota. However, their increased abundance in disease indicates a shift toward opportunistic pathogenicity, with *F. nucleatum* being the most prevalent species [22]. Furthermore, a study performed by Muchova et al. [27] showed that *F. nucleatum*-subspecies biofilm formation may help to elucidate mechanisms involved in a multi-species biofilm development and identify novel virulence factors. This may lead to novel therapeutic targets for prevention and treatment of *F. nucleatum*-mediated infections and comorbidities.

### 2.1. Virulence Factors (Adhesins, Invasions, Endotoxins)

*Fusobacterium nucleatum* has also been associated with colorectal malignancies and CVDs owing to its ability to adhere to and invade cells and evade host immune recognition [9,22]. *Fusobacterium* adhesin A (FadA), immunomodulatory protein (Fap2), and coaggregation protein (RadD) permit both host tissue colonisation and interaction with other microbial species [22]. FadA binds to E-cadherin, a cell–cell adhesion molecule present on epithelial and endothelial cells, causing disturbances in the intercellular junctions, thus allowing *F. nucleatum* to invade deeper tissues. This triggers β-catenin signalling, resulting in increased expression of inflammatory cytokines, which influences cell proliferation and apoptosis, resulting in oncogenic changes, especially important in colorectal cancer development, and the translocation of bacteria across epithelial barriers and into the bloodstream [28]. Fap2 binds to galactose-β (1–3)-N acetylgalactosamine (Gal-GalNAc) overexpressed on tumour cells, which in turn promotes tumour-specific adhesion. It also binds to TIGIT, an immune checkpoint receptor on T cells and NK cells, which stops cytotoxic activity, allowing *F. nucleatum* to evade immune response in tumours [29,30].

RadD mediates the coaggregation of other oral microbes, including *Streptococcus* species (early colonisers) and *Porphyromonas gingivalis* and *Treponema denticola* (late colonisers). This promotes the formation of multispecies biofilms, a hallmark of dental plaque and periodontal disease, highlighting the role of *F. nucleatum* in bridging early and late colonisers. These biofilms exhibit increased resistance to host immune defences and antimicrobial agents [31]. Lipopolysaccharide triggers inflammatory responses via Toll-like receptor 4 (TLR4) activation, leading to increased levels of pro-inflammatory cytokines and periodontal and systemic inflammation [25]. Proteases, such as serine protease, degrade the host protein, immune effector molecules, and extracellular matrix, promoting tissue destruction [32].

### 2.2. Colonisation of the Subgingival Plaque

Subgingival plaque refers to multispecies biofilms that form underneath the gumline (gingival margin), within the gingival sulcus, or in the periodontal pocket [33]. Colonisation begins with early colonisers that include facultative bacteria, such as *Streptococci* and *Actinomyces* species [34]. These oral bacteria adhere to the tooth surface (pellicle) via specific adhesion, which is vital for biofilm formation. Furthermore, the subgingival environment, characterised by low oxygen tension, creates favourable conditions for the increased abundance and colonisation of late colonisers, such as Bacteroidaceae species and spirochaetes [35]. *Fusobacterium nucleatum* cannot attach to the tooth pellicle efficiently on its own and instead binds to early and late colonisers, which provides a “bridge organism” for biofilm formation, thus creating an environment that favours oral disease development [36].

## 3. *Fusobacterium* and Periodontal Disease

### 3.1. Mechanisms of Periodontal Inflammation and Tissue Destruction

Periodontal disease is initiated by the build-up of pathogenic biofilms in the form of subgingival plaque on the tooth surfaces. Although the accumulation of pathogenic bacteria initiates the disease process, most tissue destruction in periodontitis results from persistent and dysregulated immune and inflammatory host responses to pathogens [37]. Chronic periodontitis is initiated by a polymicrobial infection involving key pathogens, such as *Porphyromonas gingivalis*, *Tannerella forsythia*, and *Treponeam denticola* (red complex), reinforced by bridging bacteria, such as *F. nucleatum* (orange complex) [38]. These oral bacteria possess powerful virulent factors that trigger the innate immune system, including proteases (such as gingipains) and collagenases that degrade host proteins. *Porphyromonas gingivalis* contains fimbria as a virulent factor, which facilitates adhesion and tissue colonisation [39]. The innate immune response is activated when Pathogen Associated Molecular patterns (PAMP)s are recognised by Toll-like receptors (TLRs) on neutrophils, macrophages, and epithelial cells. This initiates the recruitment of neutrophils to the gingival crevicular fluid, the release of reactive oxygen species (ROS) and proteolytic enzymes, and the production of inflammatory mediators, such as cytokines (interleukin (IL)-1β, TNF-α, IL-6), chemokines, and prostaglandins (PGE_2_). The adaptive immune response additionally contributes to the disease process, with Th1 and Th17 T-cell responses promoting chronic inflammation, while B cells and plasma cells produce antibodies, further contributing to the release of inflammatory mediators that aggravate tissue damage [40]. Connective tissue and bone destruction occurs as the disease progresses due to the activity of Matrix Metalloproteinase (MMPs), which affect collagen in the periodontal ligament and gingival connective tissue. In a healthy periodontium, inflammation is resolved once the microbial load is controlled, however in a disease state, biofilm dysbiosis sustains inflammation, creating a self-continuing cycle of tissue destruction [41].

### 3.2. Synergistic Interactions with Other Oral Pathogens

*Fusobacterium* genera, particularly *F. nucleatum*, play a key role in periodontal disease through synergistic interaction with other oral pathogens. Through these interactions, *F. nucleatum* supports the survival, nutrient exchange, and virulent expression of other periodontopathogens, thereby increasing inflammation, tissue destruction, and disease progression [42].

### 3.3. Evidence from Clinical, Animal, and Experimental Studies

Several clinical studies have provided evidence of the presence of oral bacteria, including *F. nucleatum*, within human atherosclerotic samples, using molecular analysis of endarterectomy and atherectomy specimens, eluting a direct translocation from the oral cavity to vascular tissues [43]. This finding has been supported by a larger epidemiological study where they have confirmed that chronic periodontal disease is linked to endothelial dysfunction, thickened carotid intima-media, and arterial stiffness, all of which are known risk factors for CVD [44]. Furthermore, systematic reviews have analysed evidence from patient cohorts (indicating that *F. nucleatum* DNA was frequently detected in coronary and arterial tissues) and have discussed several virulent factors, such as the adhesin FadA, which disrupts endothelial integrity and promotes vascular inflammation [9]. However, these findings should be interpreted cautiously as most studies depend on post-mortem samples and molecular detection techniques that identify bacterial DNA but cannot prove bacterial viability or active colonisation. In addition, the detected bacterial DNA may reflect passing bacteraemia originating from the oral cavity rather than persistent infection within the vascular wall [45].

Animal models using ApoE knockout mice with oral inoculation with *F. nucleatum* have reported altered systemic profiles, increased inflammatory mediators in the aortic tissues, and increased aortic plaque [9]. Other in vitro studies suggest that the presence *F. nucleatum* induces the expression of adhesion molecules and increases endothelial permeability, thereby initiating leukocyte infiltration and atherogenesis [46]. This finding has been supported by omics studies that showed *F. nucleatum* exoproteins associated with thrombus formation and plaque instability [47]. Therefore, the findings of clinical and experimental studies corroborate the hypothesis that *F. nucleatum* not only contributes to periodontal tissue destruction but may also worsen CVD through systemic dissemination, endothelial injury, and inflammatory activation.

## 4. Periodontal Disease, Systemic Inflammation, and Plaque Formation

*Fusobacterium nucleatum* has been implicated in the entire spectrum of gum disease from mild gingivitis to aggressive periodontitis with its abundance increasing parallel to the progression of inflammation and immune activation [9,25,48]. FadA, a common adhesin molecule expressed by *F. nucleatum* can bind to cadherins, specifically vascular endothelial (VE)-cadherin, present on host cells, including monocytes, erythrocytes, and natural killer cells [25,28]. This strong adhesive property allows it to penetrate cells and tissue, thereby disrupting intercellular junctions [49]. As previously discussed in Section 2.1, LPS and other bacterial components activate TLR signalling and NF-κB pathways, resulting in pro-inflammatory cytokine secretion. In periodontal disease, this results in leukocyte recruitment and sustained tissue inflammation, connecting local microbial infection to systemic vascular effects [48].

In the circulation, FadA allows the bacteria to adhere to the single layer of endothelial cells lining the vessel walls, while bacterial outer membrane vesicles (OMVs) and LPS cause endothelial dysfunction [50]. Next, LPS and OMVs activate TLRs on circulating monocytes, leading to further cytokine production and the release of monocyte chemoattract protein-1 (MCP-1). The resulting environment causes the polarisation of monocytes from the anti-inflammatory M2 phenotype to the pro-inflammatory M1 phenotype, which can penetrate the endothelial cell barrier before differentiation into macrophages [51].

LPS and other bacterial products also induce oxidative stress by reducing the production of nitric oxide, thus causing vasoconstriction. These events promote a procoagulant environment and activate endothelial cells, which upregulate expression of intercellular adhesion molecule 1 (ICAM-1) and vascular cell adhesion molecule-1 (VCAM-1). This is amplified by the binding of bacterial heat shock protein (HSP) GroEL to human HSP60 on endothelial cells. GroEL upregulates receptors for vascular endothelial growth factor (VEGF) and increases expression of tissue factor (TF) while down regulating tissue factor pathway inhibitor (TFPI) creating an environment which favours coagulation [52]. The now dysfunctional endothelium attracts more activated monocytes that cross the damaged endothelial barrier into the sub-endothelial space and become macrophages. Within the vascular intima, chronic inflammation continues to be intensified by *F. nucleatum* together with the accumulation of oxidised low-density lipids (OxLDL) [48]. The transformed macrophages upregulate scavenger receptors, such as CD36, and engulf OxLDL to form foam cells, which increase in number and form a fatty streak that surrounds a necrotic lipid core. This is the first step in the formation of an atherosclerotic plaque [9].

Vascular smooth muscle cells (VSMCs) proliferate and migrate to the vascular intima where they secrete collagen, form a fibrous cap, and stabilise the developing plaque [53]. As plaque development continues, *F. nucleatum* plays a key role in its destabilisation, thus increasing the risk of a cardiovascular event. The activated M1 macrophages secrete MMP-2 and MMP-8, which break down collagen, thereby weakening the fibrous cap while *F. nucleatum* activates caspase pathways and initiates smooth muscle apoptosis resulting in further destabilisation [48]. Continuous inflammation with TF expression, platelet activation, and initiation of coagulation pathways creates a prothrombotic environment while further instability and eventual plaque rupture results in the release of highly thrombogenic substances, thereby increasing the risk of a thrombotic event [54,55].

## 5. Cardiovascular Disease: A Microbial Perspective

Increasing evidence suggests that oral bacteria, particularly those involved in periodontitis, may contribute to CVDs through direct invasion, systemic inflammation, and molecular mimicry [56]. Atherosclerosis is a chronic inflammatory disease of the arteries characterised by the accumulation of lipids, immune cells, and fibrous elements within the vascular wall [22,57,58]. Microbial factors have emerged as important role players in this process, in addition to lipid and immune contributions [8]. Infective endocarditis represents another cardiovascular complication with microbial origins [59,60]. The condition develops when circulating bacteria colonise damaged heart valves or endocardial surfaces. Oral pathogens, especially viridans streptococci and anaerobes, such as *Fusobacterium* species, have been linked with endocarditis [61]. Poor oral hygiene and invasive dental procedures predispose individuals to bacteraemia and potential cardiac colonisation [62]. Both atherosclerosis and endocarditis underscore the role of microorganisms as triggers or accelerators of cardiovascular pathology, thereby providing a strong rationale for investigating periodontal pathogens as systemic risk factors.

### 5.1. Inflammatory Pathways Shared with Periodontitis

Chronic inflammation is a central feature of CVDs and periodontitis [63]. Both conditions are characterised by elevated levels of pro-inflammatory mediators, such as IL-6, TNF-α, and C-reactive protein (CRP) [63]. Building on the inflammatory mechanisms outlined in Section 2.1, *F. nucleatum* increases cytokine release and tissue injury, reinforcing the connection between periodontal inflammation and cardiovascular disease. Moreover, *F. nucleatum* can co-aggregate with other bacterial species in polymicrobial biofilms, which amplifies its pathogenicity and promotes chronic inflammation [22]. Chronic low-grade inflammation is a characteristic of periodontitis and may act as an amplifier of CVD. Sustained exposure to bacterial antigens and inflammatory mediators contributes to vascular injury over time. The inflammatory pathways shared between periodontitis and atherosclerosis highlight how oral pathogens, such as *F. nucleatum*, link oral disease and systemic cardiovascular complications [8].

### 5.2. The Role of Bacteraemia and Immune Cross-Reactivity

Bacteraemia represents a direct route by which periodontal pathogens influence cardiovascular health. Routine activities, such as tooth brushing, chewing, and dental procedures can introduce bacteria from periodontal pockets into the bloodstream [62]. These incidences allow organisms, such as *F. nucleatum*, to disseminate systemically. Notably, *F. nucleatum* demonstrates strong epithelial invasiveness and immune evasion capabilities, thus enabling survival in circulation and possible seeding within vascular tissues. Another proposed mechanism is immune cross-reactivity. Bacterial antigens may mimic host proteins, leading to the generation of autoantibodies that inadvertently target self-tissues. Heat shock proteins (HSPs) from *Fusobacterium* share structural homology with human HSP60, a molecule expressed on stressed endothelial cells. Antibodies directed against bacterial HSPs can cross-react with the vascular endothelium, promoting local inflammation and lesion development [64]. Furthermore, *F. nucleatum* contributes to biofilm resilience orally and within vascular tissues. As a bridging organism, it facilitates the integration of diverse bacterial species, creating stable polymicrobial biofilms that are highly resistant to immune clearance [22,47]. These multifactorial processes provide mechanistic explanations for how oral pathogens can contribute to CVD progression. The microbial aspect of CVDs adds an important layer to our understanding of pathogenesis. Oral pathogens, such as *F. nucleatum*, contribute to systemic inflammation, endothelial injury, and immune dysregulation.

## 6. Linking *Fusobacterium* to Cardiovascular Diseases

The presence of microbial signatures within arterial plaques has been a pivotal discovery in understanding the infectious component of atherosclerosis. Several studies have used culture-independent methods, such as 16S rRNA sequencing, to identify bacterial DNA, including *Fusobacterium*, in human atheromatous tissues. A landmark study by Haraszthy et al. demonstrated the detection of periodontal pathogens, including *F. nucleatum*, in atherosclerotic plaques, suggesting translocation from the oral cavity to vascular sites (Table 1) [65]. This was followed by high-resolution sequencing work by Koren et al., which identified *Fusobacterium* species in the oral microbiome and within coronary and carotid artery plaques [43]. Bacterial components, such as LPS and other virulence factors, may trigger endothelial activation, cytokine release, and macrophage infiltration, all of which contribute to plaque inflammation and instability [66]. The accumulating evidence positions *Fusobacterium* as a potential microbial contributor to the chronic inflammation that is characteristic of atherosclerosis.

**Figure 1:**
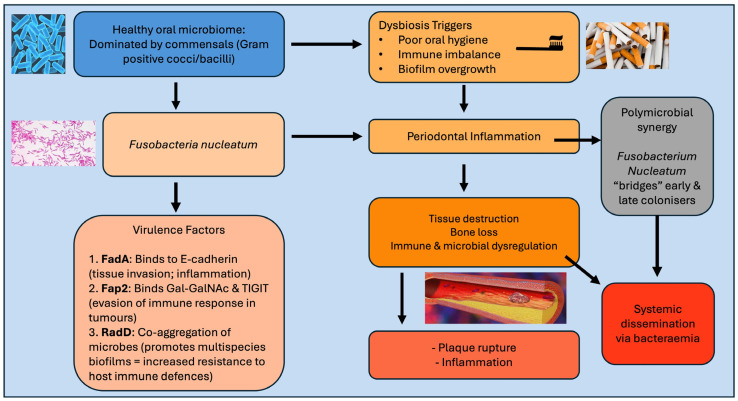
*Fusobacterium nucleatum* as a bridge between oral and cardiovascular diseases.

Epidemiological studies have been crucial in linking oral health status, microbial burden, and CVDs. Although causality remains a challenge to establish, a consistent body of evidence suggests that periodontal pathogens, including *F. nucleatum*, are associated with heightened cardiovascular risk. One of these pivotal studies was conducted by Desvarieux et al., who showed that oral bacterial burden correlated positively with carotid artery intima-media thickness (IMT), a subclinical marker of atherosclerosis [67]. Building on these findings, several studies have concluded that periodontitis is independently associated with increased CVD incidence [63,72,73]. Severe periodontitis was linked with higher rates of myocardial infarction and stroke [72,74], suggesting that microbial and inflammatory mechanisms extend beyond oral disease alone. A study by Corredor et al. compared bacterial profiles in individuals with and without coronary artery disease and showed that oral bacteria can translocate into the bloodstream and contribute to atherosclerosis [68]. Significant differences in bacterial taxa, including *F. nucleatum* and other periodontal pathogens, were observed, suggesting their possible role in vascular inflammation and plaque progression (Figure 1).

Animal models using *ApoE* knockout mice with oral inoculation with *F. nucleatum* have reported altered systemic profiles, increased inflammatory mediators in the aortic tissues, and increased aortic plaque [9]. Other *in vitro* studies suggest that the presence of *F. nucleatum* induces the expression of adhesion molecules and increases endothelial permeability, thereby initiating leukocyte infiltration and atherogenesis [46]. This finding has been supported by omics studies showing that *F. nucleatum* exoproteins are associated with thrombus formation and plaque instability [47].

Overall, the evidence linking *Fusobacterium nucleatum* to cardiovascular disease bridges multiple levels of investigation. The best supportive evidence has been derived from in vitro studies demonstrating endothelial activation, cytokine release, and immune modulation [25]. These have been confirmed in animal models showing enhanced inflammatory responses and plaque development [48,75]. In comparison, human data is mostly observational, consisting of microbial DNA detection within atherosclerotic plaques and epidemiological associations between periodontal disease and cardiovascular outcomes [44,76]. While these discoveries collectively support biological plausibility, the evidence supporting these findings remains preclinical, and direct causal relationships in humans have yet to be conclusively established [50,54,69,70,77,78].

### Shared Inflammatory and Immune Mechanisms Linking Periodontitis and CVD

Chronic inflammation is a central feature of both cardiovascular disease (CVD) and periodontitis [63]. Both these conditions are characterised by elevated levels of pro-inflammatory mediators, including IL-6, TNF-α, and C-reactive protein (CRP), which contribute to systemic inflammatory burden and vascular injury [57,63]. As discussed previously, *Fusobacterium nucleatum* plays an important role in periodontal inflammation by stimulating cytokine production and tissue destruction. In polymicrobial biofilms, *F. nucleatum* can co-aggregate with other bacterial species, enhancing its pathogenic potential and sustaining chronic inflammation [22].

In addition, molecular mimicry may also contribute to vascular damage, as heat shock proteins produced by *F. nucleatum* share structural similarities with human HSP60, potentially initiating a cross-reactive immune response that promotes endothelial injury and atherosclerosis [73]. Furthermore, *F. nucleatum* may adhere to and invade endothelial cells, increasing the expression of adhesion molecules and promoting endothelial dysfunction [46,53,74]. Together, these inflammatory and immune pathways provide a plausible biological mechanism linking periodontal infection with cardiovascular disease which makes a compelling case for further investigation.

## 7. Diagnostic and Therapeutic Implications

### 7.1. Preventive Strategies

Several studies have recognised that maintaining oral health extends beyond the prevention of oral diseases and has systemic implications. Daily mechanical plaque control, through brushing and flossing, reduces the microbial load and prevents dysbiosis [56]. Professional periodontal therapies, such as scaling and root planing, have been shown to attenuate systemic inflammation. A study by D’Aiuto et al. showed that periodontal treatment significantly lowered circulating markers of inflammation, namely CRP and IL-6 [71].

Modulation of the oral microbiome is an emerging strategy. Probiotics, such as *Lactobacillus* and *Streptococcus salivarius*, can competitively inhibit pathogenic species, including *F. nucleatum* [79]. Preliminary studies indicate that probiotic interventions may reduce periodontal inflammation and suppress pathogenic biofilms. By restoring the microbial balance, probiotics may mitigate systemic effects of oral dysbiosis; however, further clinical trials are needed to corroborate these findings. Incorporating oral health assessments into CVD risk stratification may improve the early identification of high-risk patients, especially those with severe periodontitis and a family history of CVD. From a public health standpoint, integrating dental care into chronic disease management programmes may reduce the overall burden of CVDs [12,62]. Routine medical evaluations should include oral health status, emphasising the importance of interdisciplinary collaboration.

### 7.2. Potential Targets for Antimicrobial or Anti-Inflammatory Therapy

Therapeutic strategies against *F. nucleatum* aim to reduce pathogen burden, reduce systemic inflammation, and restore host-microbiome balance. Several promising avenues are under investigation, including antimicrobials, natural compounds and phytochemicals, host modulation therapy, immunotherapy, and vaccines.

Antimicrobial therapy in periodontitis, including local application of chlorhexidine or minocycline gels, can reduce *F. nucleatum* colonisation, while antibiotics, such as amoxicillin and metronidazole, can be used in refractory cases. However, there are concerns regarding antimicrobial resistance and microbiome disruption [80,81]. Future efforts should focus on narrow-spectrum agents that specifically target *F. nucleatum* while sparing commensals. Plant-derived agents, such as curcumin, resveratrol, and catechins from green tea, are known to exhibit antimicrobial and anti-inflammatory effects [82]. Studies have shown that these compounds inhibit biofilm formation by *F. nucleatum* and attenuate the release of inflammatory mediators, thus making these compounds attractive candidates for adjunctive therapy since they may induce fewer side effects than some antibiotics [82,83,84].

**Figure 2:**
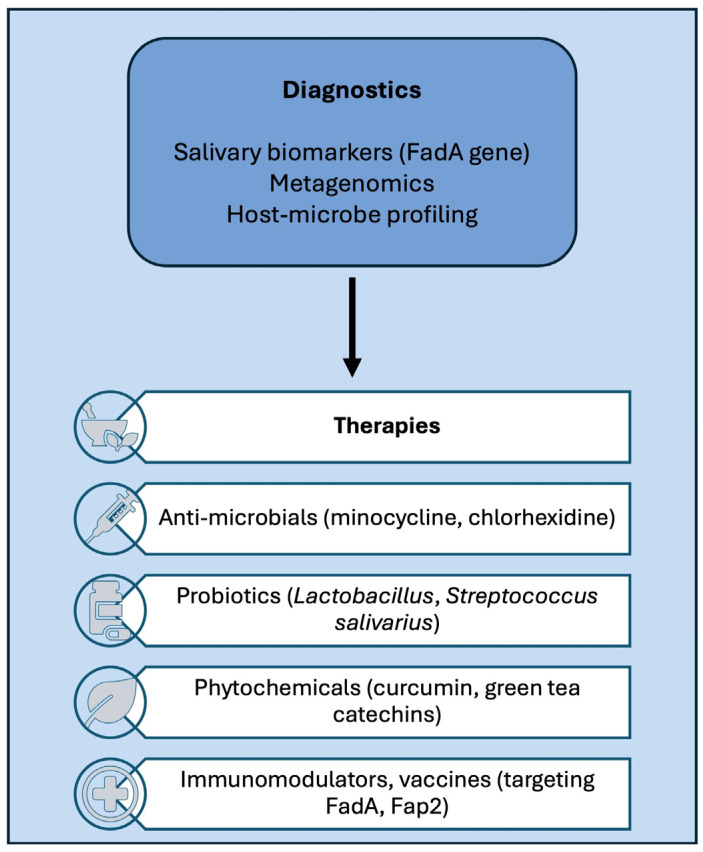
Diagnostic and therapeutic options to combat *Fusobacterium nucleatum*.

Another option is to target the inflammatory response rather than the pathogen. Inhibitors of NF-κB and cytokine signalling have shown promise in reducing inflammation. Statins are widely prescribed for hyperlipidaemia, but may produce anti-inflammatory and antibacterial effects, which may aid in reducing periodontitis and improving vascular outcomes [85]. Although still in the experimental stages, vaccines targeting *F. nucleatum* adhesins or outer membrane proteins may prevent colonisation and systemic dissemination [86]. Thus, it is important to promote the integration of antimicrobial, anti-inflammatory, and host-directed methods to effectively treat *F. nucleatum* colonisation and the associated diseases (Figure 2). However, current therapeutic strategies remain limited. Studies on probiotics show considerable heterogeneity in strains, doses, and reported outcomes, while antibiotic therapy has not demonstrated clear benefits for cardiovascular disease prevention [87]. Vaccines targeting periodontal pathogens, such as *F. nucleatum*, remain largely experimental [25].

## 8. Future Directions

### 8.1. Need for Longitudinal and Interventional Studies

While recent studies provide evidence of the presence of *F. nucleatum* in endothelial dysfunction and atherogenesis, most data are derived from cross-sectional and in vitro research [88]. In a prospective study, researchers found that *F. nucleatum* mediates a high risk in chronic heart disease [89]. However long term, prospective cohorts are required to determine how the presence of *F. nucleatum* influences CVD. Interventional trials targeting *F. nucleatum* through antimicrobial therapies, oral hygiene intervention, or microbiome modulation could clarify whether reducing burden translates into measurable improvement in CVD health and reduced atherosclerotic risk [90]. It remains unclear how *F. nucleatum* interacts with other oral and gut microbiota to influence systemic inflammation and vascular damage, therefore metagenomics and multi-omics may assist in evaluating whether these effects are synergistic or strain specific [9].

### 8.2. Role of Host Genetics and Microbiome Diversity

*Fusobacterium nucleatum* plays a key role as a structural and metabolic hub in the oral microbiome, linking early and late colonisers, which contributes to the progression of periodontal dysbiosis and inflammation [9]. The human genetic background also plays a crucial part in shaping the immune response to *F. nucleatum*. The microorganism produces virulent factors, such as LPS, adhesins, and proteases, that interact with host immune receptors. Recognition of these microbial products is facilitated by pattern recognition receptors (e.g., TLR2, TLR4, and NOD2-like receptors). Polymorphisms in genes may alter recognition of microbial ligands and the magnitude of the inflammatory response. For instance, certain TLR4 polymorphisms decrease responsiveness to LPS, allowing perseverance of *F. nucleatum*, while others lead to hyper-responsiveness and exaggerated inflammation, resulting in tissue destruction [91,92].

### 8.3. Potential for Precision Medicine Approaches

Traditional laboratory approaches, such as culture, lack sensitivity and specificity, while precision-based techniques provide precise detection, patient-specific risk assessment, and personalises interventions [93]. Current research supports the use of salivary biomarkers in point of care testing technologies to enhance early diagnosis and effective personalised treatment [94].

### 8.4. Metagenomic and Metatranscriptomic Sequencing

Next-generation sequencing (NGS) enables precise identification of oral pathogens, such as *F. nucleatum*, within complex microbial communities. Not only does NGS recognise virulent strains, but it can quantify the bacterial abundance and distinguish multiple species from each other. This provides clinicians with population-level-data regarding the genome of pathogens, providing valuable insights for disease management and systemic risk [95].

### 8.5. Biomarker Discovery

The use of biomarkers is vital for precision medicine and in correlating health and disease states. The genetic markers *fadA* in *F. nucleatum* has been linked to oral heath, particularly in colorectal cancers [96,97]. Therefore, early detection may provide predictive and prognostic value for periodontal disease and other systemic comorbidities This has been supported by a systematic review that focused on the biological role of *F. nucleatum* in the diagnosis and prognosis of gastrointestinal malignancies [98].

### 8.6. Host-Microbe Interaction Profiling

Host-microbe interaction profiling involves the analysis of molecular, cellular, and systematic responses that occur when a microorganism interacts with a host. It involves both microbial (adhesion, toxins, metabolites) and host (gene expression, immune signalling, and barrier function) responses. Differences in immune responses to *F. nucleatum* can greatly influence disease outcomes. Furthermore, precision profiling of host factors, such as inflammatory cytokine signatures and Toll-like receptor activation, allows for the identification of individuals at an increased risk of exaggerated systemic inflammatory responses associated with oral microbial colonisation [9]. Precision medicine using oral bacteria offers prospective groundwork in both oral and systemic health. These applications integrate genomics, biomarkers, host responses, and digital health approaches, which clinicians may use to personalise the treatment of diseases associated with *F. nucleatum*.

Despite current evidence supporting the link between *F. nucleatum*, periodontal disease, and cardiovascular disease, several limitations have been highlighted. For instance, much of the mechanistic insight is derived from in vitro and animal models, while human data remains largely observational and associative. The presence of microbial DNA within atherosclerosis plaque does not provide direct evidence of microbial viability or causality. Furthermore, periodontitis is a complex multifactorial inflammatory disease driven by polymicrobial dysbiosis and host immune dysregulation. The heterogeneity of study design, microbial detection methods, and cardiovascular endpoints further complicates direct comparison across studies. Further longitudinal and interventional research is recommended to clarify causal relationships, therapeutic implications, and clinical relevance.

## 9. Conclusions

Growing evidence has highlighted the compelling link between *F. nucleatum* and other *Fusobacterium* species in the development of both periodontal and cardiovascular diseases [9]. *Fusobacterium* in the oral cavity triggers local inflammation, leading to possible tissue destruction and systemic inflammation, which has been highlighted as a well-established risk factor for CVDs [48,86]. Thus, *F. nucleatum* has been identified as an opportunistic pathogen that plays a key role in the interaction between other bacteria and the host in many infectious diseases. The establishment of omics technology has provided a method to closely monitor the presence of oral bacteria and their associations in other diseases [29,50,52,99,100]. From a clinical perspective, these findings underline the vital importance of early detection and management of periodontal disease, not only to maintain oral health but also to reduce the development of CVDs. From a public health perspective, promoting oral health education and CVD prevention campaigns may have a greater impact in reducing the prevalence of periodontal disease [11]. Therefore, we recommend longitudinal studies to investigate mechanistic pathways and potential interventional approaches to improve patient outcomes.

## Figures and Tables

**Table 1:** Evidence linking *Fusobacterium nucleatum* to atherosclerosis and cardiovascular disease.

Evidence Type	Key Findings	Published Studies	Strengths	Limitations
Detection of microbial DNA in atherosclerotic plaques	Culture-independent techniques (e.g., 16S rRNA sequencing) have detected bacterial DNA in atheromatous plaques, suggesting possible translocation from the oral cavity to vascular tissues.	Haraszthy et al. [65]; Koren et al. [43]	Direct evidence of microbial signatures within vascular lesions; supports biological plausibility of oral–vascular microbial translocation.	Detection of DNA does not confirm bacterial viability or causality; contamination and transient bacteraemia cannot be excluded.
Epidemiological and observational studies	Associations have been reported between periodontal disease, oral microbial burden, and cardiovascular outcomes, such as increased carotid intima-media thickness (IMT), myocardial infarction, and stroke.	Desvarieux et al. [67]; Corredor et al. [68]; other epidemiological studies, including Koren et al. [43], Wang et al. [66], Chew et al. [69], and Ou et al. [70].	Large population-based data; demonstrates consistent associations between oral health and cardiovascular risk.	Observational design limits causal inference. Confounding factors (e.g., smoking, diabetes, socioeconomic status) may influence outcomes.
Animal models	Oral inoculation of *F. nucleatum* in ApoE knockout mice has been associated with increased systemic inflammation, elevated inflammatory mediators in aortic tissues, and increased atherosclerotic plaque formation.	ApoE mouse studies [10].	Allows controlled investigation of mechanistic pathways and disease progression.	Animal physiology does not fully replicate human cardiovascular disease; experimental exposure may exceed natural infection levels.
*In vitro* mechanistic studies	*F. nucleatum* exposure induces endothelial activation, adhesion molecule expression, cytokine production, and increased endothelial permeability, facilitating leukocyte infiltration and early atherogenesis.	Cellular studies [71] and omics-based exoprotein studies [72].	Provides detailed insight into molecular mechanisms and host–pathogen interactions.	Simplified experimental systems lack the complexity of the human immune and vascular environment.

## Data Availability

No new data were created or analyzed in this study.
